# Evaluating Indirect Economic Losses from Flooding Using Input–Output Analysis: An Application to China’s Jiangxi Province

**DOI:** 10.3390/ijerph20054509

**Published:** 2023-03-03

**Authors:** Yanfang Lyu, Yun Xiang, Dong Wang

**Affiliations:** 1 School of Statistics, Huaqiao University, Xiamen 361021, China; 2 School of Economics and Finance, Huaqiao University, Quanzhou 362021, China; 3 School of Business, Minnan Normal University, Zhangzhou 363000, China

**Keywords:** indirect economic losses, input–output analysis, industrial linkages, spillover effect, structural decomposition analysis

## Abstract

Quantifying total economic impacts of flood disaster in a timely manner is essential for flood risk management and sustainable economic growth. This study takes the flood disaster in China’s Jiangxi province during the flood season in 2020 as an example, and exploits the input–output method to analyze indirect economic impacts caused by the agricultural direct economic loss. Based on regional IO data and MRIO data, a multi-dimensional econometric analysis was undertaken in terms of inter-regional, multi-regional, and structural decomposition of indirect economic losses. Our study reveals that the indirect economic losses caused by the agricultural sector in other sectors in Jiangxi province were 2.08 times the direct economic losses, of which the manufacturing sector suffered the worst, accounting for 70.11% of the total indirect economic losses. In addition, in terms of demand side and supply side indirect losses, the manufacturing and construction industries were found to be more vulnerable than other industries, and the flood disaster caused the largest indirect economic loss in eastern China. Besides, the supply side losses were significantly higher than the demand side losses, highlighting that the agricultural sector has strong spillover effects on the supply side. Moreover, based on the MRIO data of the years 2012 and 2015, dynamic structural decomposition analysis was undertaken, which showed that changes in the distributional structure appear to be influential in the evaluation of indirect economic losses. The findings highlight the spatial and sectoral heterogeneity of indirect economic losses caused by floods, and have significant implications for disaster mitigation and recovery strategies.

## 1. Introduction

### 1.1. Background

As a result of global climate change, the frequency and intensity of natural disasters have increased significantly in recent years. Among all natural disasters, floods are the most common in terms of incidence, affected population, area, and related economic losses [1,2]. As flood disasters have negative effects on real GDP, residents’ income, consumption, and several other macroeconomic indicators [3], quantifying the spatial distribution and corresponding effects after extreme shocks is critical for both post-disaster reconstruction and sustainable development objectives [4]. Flood exposure will further increase with the intensification of the global hydrological cycle, posing a serious threat to future generations [5,6,7]. Therefore, it is imperative to assess flood risks against the background of global climate change and socio-economic sustainable development, with the purpose of supporting post-disaster recovery and adaptation strategies.

The assessment of economic losses after a disaster can contribute to disaster prevention and mitigation, increase the practicability of disaster assistance, and strengthen public awareness of the effects of disasters [8]. However, limited exploration has been conducted of the socio-economic impacts of floods in a warming world [5,7]. Generally, economic losses can be classified into two sorts: direct and indirect [9]. Direct economic losses usually refer to the physical damage caused by natural disasters [10,11]. During or soon after flooding, direct economic losses are assessed in a timely manner. Because they reflect the monetary value of the destruction of physical assets within the affected area, direct economic losses are mostly tangible and easy to be quantified [12,13,14]. Resulting from direct economic losses, indirect impacts capture the short-term and long-term economic losses in economic production, consumption, and any related economic recovery paths [15]. Propagating through the interruptions of supply chains and industrial linkages, indirect economic losses may not only incur within the area exposed to the flood disaster, but also arise outside the area. In addition, the indirect damage may have the two characteristics of regional heterogeneity and temporal dynamics in the networked economy [16,17]. Regarding the magnitude of indirect economic losses, it may even be greater than that of direct economic losses [18,19]. Moreover, indirect losses are always intangible [14], and existing research on measuring them mostly comprises qualitative descriptions [19]; thus, our study aims at pursuing further advances in this regard.

### 1.2. Literature Review

Compared with any other industry, agricultural production is much more affected by the environment and climate [20]. Extreme changes in rainfall patterns have serious impacts on crop production [21]. In developing countries, in particular, significantly more people depend on the agricultural sector for sustenance and their ability to adapt to climate uncertainty is also weak [22,23]. As it relates closely to grain supplies, developing climate-resilient crop varieties, so as to relieve the negative effects of climate change on agricultural production, is thus essential [24]. Human capital, physical capital, natural capital, financial capital, and social capital are crucial reserves for rural affected households to better cope with and adapt to floods, because these households are exposed to increasing floods risks [25]. In addition, strengthening the construction of flood control facilities, for example, the installation of storage and drainage equipment, is beneficial to making agricultural activities more sustainable [20].

Estimating the consequences of such floods on local and regional areas is essential to improving flood prevention, emergency responses, and recovery strategies [26]. According to existing research, both the direct affected area and the remedies for reducing direct economic losses correspond to a clear target object due to its tangible attributes. However, as floods incur local agricultural losses, how other sectors are indirectly involved both within and outside the affected area, determining the size of the overall economic losses is not so intuitive. In this regard, it is necessary to pay more attention to the intermediate diffusion paths of direct economic losses. Thus, we place more emphasis on measuring indirect economic losses caused by floods. In the field of flooding and indirect economic losses, Table 1 below lists the relevant studies involving the main models.

For the evaluation of indirect economic losses of natural disasters, the representative models mainly include the input–output (IO) model [18,27,28,29], production function model, computable general equilibrium (CGE) model [30,31,32], and social accounting matrix model, of which IO and CGE models are applied more commonly. IO models are advantageous because of their simplicity and ability to consider the industrial interdependencies, whereas CGE models are characterized by their non-linearity and flexibility in including supply side effects [13,33]. Regarding practical application, CGE models require many parameters to be calibrated and can easily deviate from reality. Traditional IO models are limited to undertaking static analysis, and can easily overstate the impacts of non-affected regions because they do not consider substitution possibilities between imports from different regions [3,33]. To overcome these shortcomings in traditional IO models, there have been some developments to improve the measurement, for example, reflecting the conditions of a poor economy caused by disaster shocks [34], considering the influence of the time factor [35], and constructing an adaptive regional IO model [18,36]. However, these modifications mainly focus on indirect economic losses in the disaster area. In the view of this, some researchers have applied multi-regional input–output (MRIO) to take into account production technologies, industrial chains, and substitution effects [37,38]. Although these models have provided some frameworks to estimate the overall economic impacts, the information about the mechanism and magnitude of indirect losses is still limited.

**Table 1:** Overview of models measuring indirect economic losses of disasters.

Research	Model	Application and Characteristics
Santos and Haimes (2004) [34]	IO model	Describe how terrorism-induced perturbations propagate; recognize the affected sectors at the regional scale
Rose and Liao (2005) [13]	CGE model	Estimate the economic impacts of a disruption to the Portland Metropolitan Water System; require many parameters to be calibrated
Hallegatte (2008) [18]	Adaptive regional IO model	Simulate the response of the economy of Louisiana to Hurricane Katrina; consider adaptive behaviors such as substitution
Hallegatte (2014) [39]	Inventory-ARIO model	Identify which bottlenecks are responsible for output losses during two periods after Hurricane Katrina; consider the roles of inventories
Carrera et al. (2015) [33]	CGE model	Assess indirect economic impacts of the destructive Po river flood in Italy; use a regionally calibrated version of a global CGE model
Koks and Thissen (2016) [37]	MRIO model	Assess economic impacts of three floods in Rotterdam, the Netherlands; combine linear programming and IO model, consider production technologies and supply side constraints
Mendoza-Tinoco et al. (2017) [36]	IO model	Assess economic impacts of the 2007 summer floods in the region of Yorkshire and the Humber; introduce flood footprint concept
Wang et al. (2021) [40]	Adaptive inter-regional IO model	Estimate indirect economic impacts of sequential typhoons (Utor, Usagi, and Fitow); track the dynamic adaptive process of economic system

### 1.3. Research Objective, Originality, and Contribution

As mentioned above, few studies have dynamically measured impacts of flood disasters in terms of both sector and spatial dimensions, which is one of the major gaps in the current literature. In this paper, we regard the extreme flood disaster that occurred in China’s Jiangxi province in 2020 as the application scenario, take advantage of a combined methodology, and apply a relatively more flexible IO method to quantitatively evaluate indirect economic losses. Our findings can strengthen the understanding of the overall economic effects of the floods on Jiangxi and the remainder of China.

On the basis of existing research, our study contributes to the literature in two ways: (1) In view of the object and scope of flood impacts, this paper conducts a more comprehensive analysis from intra-regional to inter-regional perspectives, and from static to dynamic perspectives, based on the availability of MRIO data. We analyze the indirect economic losses from the perspective of both the demand and the supply side. Adopting the structural decomposition technology, we recognize that the internal causes of indirect economic losses are through spillover effects. Our theoretical framework of dynamic structural decomposition analysis can be expanded to other areas according to specific research needs. Thus, our work allows a more exhaustive comprehension. (2) The methodology can make a rapid preliminary assessment of indirect economic losses caused by floods, which improves the timeliness of loss recognition, and provides some guidance for post-disaster recovery and reconstruction in different departments and regions. Moreover, this study reveals that the agricultural sector is vulnerable to natural disasters, and has remarkable ripple effects on other sectors in the networked economy. Industrial linkages are playing an increasing role in sustainable economic development.

## 2. Methods

**Figure 1:**
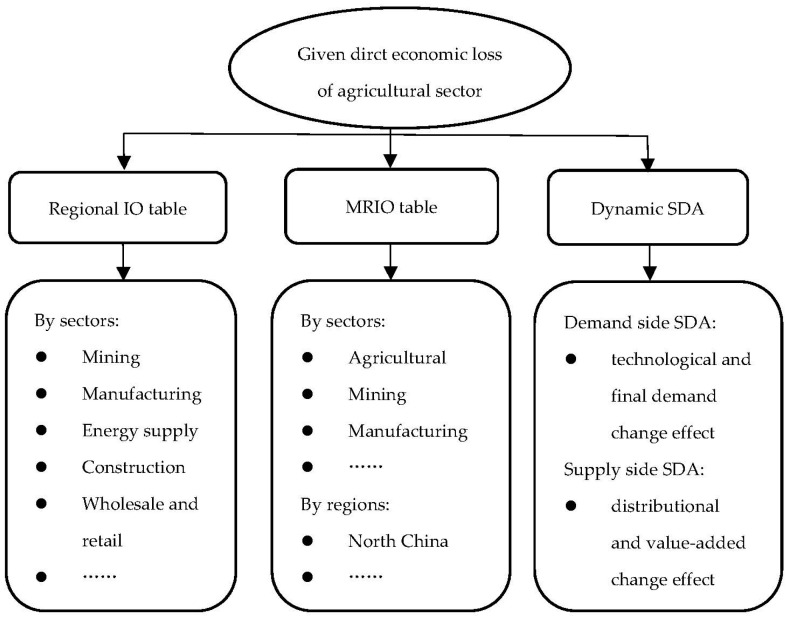
A brief exhibition of the methodology to calculate indirect economic losses.

We begin with a brief framework that illustrates the following analytical procedures so as to facilitate a better understanding of the methodology. Figure 1 shows the key elements of the framework. Our study takes the agricultural sector as the most vulnerable sector and assumes that the direct economic loss is given, as we place more emphasis on the evaluation of indirect economic losses. The regional IO table is used to calculate the indirect impacts on other economic sectors within the affected area. To reflect the diffusion trajectory of the direct economic losses across other regions and sectors, the latest available MRIO table was used. Moreover, in the dynamic structural decomposition analysis (SDA), we used two MRIO tables to compare the indirect economic losses and explain their underlying causes.

### 2.1. Descriptions of Regional IO Model

#### 2.1.1. Assess Indirect Economic Losses on the Demand Side

As the most basic IO model, the static physical IO model was first proposed by Leontief in 1936. The regional IO model reflects the input and output relationship between social and economic sectors of a specific region in a given period. The equilibrium relationship of sector *i* can be represented horizontally as:

(1) $$\sum_{j=1}^{n}z_{ij}+Y_{i}=Q_{i}\;\left(i=1,2,\cdots ,n\right)$$

where *z_ij_* represents the direct consumption of sector *i*’s products by sector *j*, *Y_i_* and *Q_i_* represent final demand and total output of sector *i*, respectively.

The definition of direct consumption coefficient is:

(2) $$a_{ij}=\frac{z_{ij}}{Q_{j}}\;\left(i=1,2,\cdots ,n\right)$$

Thus, we can organize and express the above relationship using a matrix *AQ* + *Y* = *Q*; therefore:

(3) $$Q={\left(I-A\right)}^{-1}Y$$

where *I* represents an identity matrix, ${\left(I-A\right)}^{-1}$ is the Leontief inverse matrix, which is recorded as $\tilde{B}$, and the elements are expressed by ${\tilde{b}}_{ij}$. Formula (3) is transformed into incremental form:

(4) $$∆Q={\left(I-A\right)}^{-1}∆Y$$

Flood damages per economic sector are essential input data for further calculating inter-regional indirect economic impacts [41]. Since the direct economic loss of the agricultural sector includes not only the loss of the final product, but also the reduction in intermediate consumption of agricultural products, the direct economic loss of the agricultural sector is its total output loss, $∆Q_{1}$. In terms of horizontal relationships in the IO table, the damage of the agricultural sector will result in losses in sectors that provide agricultural production materials, so the indirect economic losses calculated horizontally mainly reflect the demand side loss. It is assumed that the final demand of other sectors does not change, i.e., $∆Y_{j}=0\;(j\neq 1)$.

According to Formula (4), the change in the total output can be described as the following column vector format:

(5) $$\left[\begin{array}{c} ∆Q_{1}\\ ∆Q_{2}\\ \vdots \\ ∆Q_{n} \end{array}\right]=\left[\begin{array}{ccc} {\tilde{b}}_{11} & \cdots & {\tilde{b}}_{1n}\\ \vdots & \ddots & \vdots \\ {\tilde{b}}_{n1} & \cdots & {\tilde{b}}_{nn} \end{array}\right]*\left[\begin{array}{c} ∆Y_{1}\\ 0\\ \vdots \\ 0 \end{array}\right]$$

Therefore, the final product loss of the agricultural sector can be obtained first, i.e.,

(6) $$∆Y_{1}=\frac{∆Q_{1}}{{\tilde{b}}_{11}}$$

Then, for the demand side, the total indirect output loss of sectors other than the agricultural sector within the local region is obtained, as Formula (7) demonstrates below:

(7) $$∆Q_{i}=\frac{{\tilde{b}}_{i1}∆Q_{1}}{{\tilde{b}}_{11}}$$

In order to differentiate correlative relations within industries from those among industries, we define the spillover effect I as $S_{1i}=\sum_{j,j\neq i}^{n}{\tilde{b}}_{ij}$, and the spillover effect II as $S_{2i}=\sum_{i,j\neq i}^{n}{\tilde{b}}_{ij}$. Similar to the meaning of the response coefficient and influence coefficient in the IO method, spillover effect I indicates the degree of demand induced in industry *i* when other industries increase one unit of final product, whereas spillover effect II indicates the impact of one unit of final demand of industry *j* on other industries. Spillover effects I and II are isolated within the industry influence, constituting an effective improvement in the traditional response coefficient and influence coefficient.

#### 2.1.2. Assess Indirect Economic Losses on the Supply Side

Similarly, when the agricultural sector is affected, those sectors with agricultural products as inputs for production will suffer as a result, thus generating indirect economic losses on the supply side. The vertical balance relationship of the IO table can be expressed as:

(8) $$\sum_{i=1}^{n}z_{ij}+V_{j}=Q_{j}\;\left(j=1,2,\cdots ,n\right)$$

The distribution coefficient is used to reflect the allocation of products across sectors; the expression is:

(9) $$h_{ij}=\frac{z_{ij}}{Q_{i}}\;(i,j=1,2,\cdots ,n)$$

We can obtain the matrix expression represented as *H*′*Q* + *V* = *Q*. After a simple matrix transformation, the following equation is available:

(10) $$Q={\left(I-H^{\prime }\right)}^{-1}V$$

Transfer of Formula (10) into incremental form yields:

(11) $$∆Q={\left(I-H^{\prime }\right)}^{-1}∆V$$

The elements of the matrix (*I* − *H*′)^−1^ are represented by *g_ij_*. When calculating the indirect economic losses on the supply side, the value-added loss of the agricultural sector is calculated first, which is similar to the method applied on the demand side:

(12) $${∆V}_{1}=\frac{{∆Q}_{1}}{g_{11}}$$

Regarding the supply side, the change in the indirect economic losses except those of the agricultural sector is thus obtained, i.e.,

(13) $${∆Q}_{j}=\frac{{g_{i1}∆Q}_{1}}{g_{11}}\;(j\neq 1)$$

Similar to the expression of the demand side spillover effect, the supply side spillover effects I and II are defined respectively, i.e., $S_{1i}=\sum_{j,j\neq i}^{n}g_{ij}$, $S_{2i}=\sum_{i,j\neq i}^{n}g_{ij}$.

### 2.2. Descriptions of MRIO Model

In order to systematically and comprehensively reflect the impact of the affected sector on other regions and sectors, the MRIO model is used as an effective research tool. In the basic structure of the MRIO model, $z_{ij}^{pq}$ indicates the direct consumption of region *q*’s *j*-sector products by region *p*’s *i*-sector products, or the direct distribution of region *p*’s *i*-sector products to region *q*’s *j*-sector products; $Y_{i}^{pq}$ indicates the final use of region *p*’s *i*-sector for region *q*; $Q_{i}^{p}$ represents the total output of region *p*’s *i*-sector.

Taking the calculation of indirect economic losses on the demand side as an example, assuming the impacted area is *i*, then the direct economic loss of the agricultural sector can be expressed as ${∆Q}_{1}^{i}$; thus, the total output change can be expressed as shown in Formula (14):

(14) $$\left[\begin{array}{c} \Delta Q_{1}^{1}\\ \vdots \\ \Delta Q_{n}^{1}\\ \vdots \\ \Delta Q_{1}^{i}\\ \vdots \\ \Delta Q_{n}^{i}\\ \vdots \\ \Delta Q_{1}^{m}\\ \vdots \\ \Delta Q_{n}^{m} \end{array}\right]=\left[\begin{array}{ccccccccccc} {\tilde{b}}_{11}^{11} & \cdots & {\tilde{b}}_{1n}^{11} & \cdots & {\tilde{b}}_{11}^{1i} & \cdots & {\tilde{b}}_{1n}^{1i} & \cdots & {\tilde{b}}_{11}^{1m} & \cdots & {\tilde{b}}_{1n}^{1m}\\ \vdots & \vdots & \vdots & \vdots & \vdots & \vdots & \vdots & \vdots & \vdots & \vdots & \vdots \\ {\tilde{b}}_{n1}^{11} & \cdots & {\tilde{b}}_{nn}^{11} & \cdots & {\tilde{b}}_{n1}^{1i} & \cdots & {\tilde{b}}_{nn}^{1i} & \cdots & {\tilde{b}}_{n1}^{1m} & \cdots & {\tilde{b}}_{nn}^{1m}\\ \vdots & \vdots & \vdots & \vdots & \vdots & \vdots & \vdots & \vdots & \vdots & \vdots & \vdots \\ {\tilde{b}}_{11}^{i1} & \cdots & {\tilde{b}}_{1n}^{i1} & \cdots & {\tilde{b}}_{11}^{ii} & \cdots & {\tilde{b}}_{1n}^{ii} & \cdots & {\tilde{b}}_{11}^{im} & \cdots & {\tilde{b}}_{1n}^{im}\\ \vdots & \vdots & \vdots & \vdots & \vdots & \vdots & \vdots & \vdots & \vdots & \vdots & \vdots \\ {\tilde{b}}_{n1}^{i1} & \cdots & {\tilde{b}}_{nn}^{i1} & \cdots & {\tilde{b}}_{n1}^{ii} & \cdots & {\tilde{b}}_{nn}^{ii} & \cdots & {\tilde{b}}_{n1}^{im} & \cdots & {\tilde{b}}_{nn}^{im}\\ \vdots & \vdots & \vdots & \vdots & \vdots & \vdots & \vdots & \vdots & \vdots & \vdots & \vdots \\ {\tilde{b}}_{11}^{m1} & \cdots & {\tilde{b}}_{1n}^{m1} & \cdots & {\tilde{b}}_{11}^{mi} & \cdots & {\tilde{b}}_{1n}^{mi} & \cdots & {\tilde{b}}_{11}^{mm} & \cdots & {\tilde{b}}_{1n}^{mm}\\ \vdots & \vdots & \vdots & \vdots & \vdots & \vdots & \vdots & \vdots & \vdots & \vdots & \vdots \\ {\tilde{b}}_{n1}^{m1} & \cdots & {\tilde{b}}_{nn}^{m1} & \cdots & {\tilde{b}}_{n1}^{mi} & \cdots & {\tilde{b}}_{nn}^{mi} & \cdots & {\tilde{b}}_{n1}^{mm} & \cdots & {\tilde{b}}_{nn}^{mm} \end{array}\right]*\left[\begin{array}{c} 0\\ \vdots \\ 0\\ \vdots \\ \Delta Y_{1}^{i}\\ \vdots \\ 0\\ \vdots \\ 0\\ \vdots \\ 0 \end{array}\right]$$

The change in the total output of region *p*’s *i*-sector can be expressed as:

(15) $${∆Q}_{i}^{p}=\frac{{\tilde{b}}_{i1}^{p1}*{∆Q}_{1}^{i}}{{\tilde{b}}_{11}^{ii}}$$

For region p’s *i*-sector, the demand side spillover effects I and II are defined respectively as $S_{1i}^{p}=\sum_{j,j\neq i}^{n}\sum_{q,q\neq p}^{m}{\tilde{b}}_{ij}^{pq}$, $S_{2i}^{p}=\sum_{i,j\neq i}^{n}\sum_{p,p\neq q}^{m}{\tilde{b}}_{ij}^{pq}$.

Similarly, for the calculation of indirect economic losses on the supply side, we set the elements in the matrix (*I* − *H*′)^−1^ as $g_{ij}^{pq}$; then, the total output change in region *q*’s *j*-sector can be expressed as:

(16) $${∆Q}_{i}^{p}=\frac{g_{j1}^{q1}*{∆Q}_{1}^{i}}{g_{11}^{ii}}\;(q=1,2,\cdots ,m;j=2,3,\cdots ,n)$$

The spillover effects I and II for region *q*’s *j*-sector *j* can be expressed respectively as $S_{1j}^{q}=\sum_{j,j\neq i}^{n}\sum_{q,q\neq p}^{m}g_{ij}^{pq}$, $S_{2j}^{q}=\sum_{i,i\neq j}^{n}\sum_{p,p\neq q}^{m}g_{ij}^{pq}$.

### 2.3. Descriptions of the Structural Decomposition Method

The calculation of the indirect economic losses using the MRIO model described above is based on one yearly MRIO table that is the latest available. Structural decomposition technology can be used as an effective analytical tool to reflect the main factors that cause changes in indirect economic losses. In the view of this, we use two MRIO tables to calculate indirect economic losses so as to compare the changes. Furthermore, we analyze the reasons for the difference from the demand side and the supply side.

From the demand side perspective, define ∆X as the difference in changes in indirect economic losses calculated from the two annual MRIO tables, i.e.,

(17) $$∆X={∆Q}_{1}-{∆Q}_{0}={\tilde{B}}_{1}{∆Y}_{1}-{\tilde{B}}_{0}{∆Y}_{0}$$

Formula (17) can be further expressed by referring to the treatment of two polar decomposition averages in structural decomposition analysis:

(18) $$∆X=\frac{1}{2}\left(∆\tilde{B}\right)\left({∆Y}_{1}+{∆Y}_{0}\right)+\frac{1}{2}({\tilde{B}}_{0}+{\tilde{B}}_{1})({∆Y}_{1}-{∆Y}_{0})$$

The first item at the right-hand side of Equation (18) represents the effect of economic and technological changes, and the latter represents the effect of changes in final demand.

Similarly, from the supply side perspective, we can obtain:

(19) $$∆X={∆Q}_{1}-{∆Q}_{0}={\left(I-H_{1}^{\prime }\right)}^{-1}{∆V}_{1}-{\left(I-H_{0}^{\prime }\right)}^{-1}{∆V}_{0}$$

Through structural decomposition, Formula (19) can be further organized as follows:

(20) $$∆X=\frac{1}{2}\left[{\left(I-H_{1}^{\prime }\right)}^{-1}-{\left(I-H_{0}^{\prime }\right)}^{-1}\right]\left({∆V}_{1}+{∆V}_{0}\right)+\frac{1}{2}\left[{\left(I-H_{1}^{\prime }\right)}^{-1}+{\left(I-H_{0}^{\prime }\right)}^{-1}\right]\left({∆V}_{1}-{∆V}_{0}\right)$$

With reference to the formulation of Equation (18), the first item on the right-hand side of Equation (20) indicates the effect of changes in distribution structure, and the latter indicates the effect of value-added changes.

In summary, Equations (7) and (13) are mainly used for calculations of indirect economic losses within the affected area, whereas Equations (15) and (16) are applied to assess indirect economic losses from the perspective of inter-regional evaluation. Moreover, Equations (18) and (20) are used to give reasonable explanations for the underlying influential elements in the evaluation of indirect economic losses.

## 3. Case study

### 3.1. Data Sources

In the summer of 2020, heavy rainfall continued to occur in southern China and caused flood disasters in many places. From 1 June to 7 July, the average precipitation in the Yangtze River Basin was as high as 346.9 mm, which exceeded the average precipitation level in 1998 and became the second highest in the same period since 1961. Among the provinces and cities experiencing serious flooding in the Yangtze River Basin, Jiangxi was the first province to upgrade the flood emergency response to the highest level, i.e., level 1. This was the first time that this level had been reached in Jiangxi province since 2010. The flood disaster occurred in China’s Jiangxi province from 6 July to 22 July in 2020, significantly disturbing the intra-regional and inter-regional economic system. Thus, we selected it as an appropriate case.

This paper mainly uses the flood disaster information and input–output table. In terms of the disaster data, according to the release of Jiangxi Provincial Water Resources Department, the direct economic loss of the province’s agricultural sector due to the flooding was CNY 10.07 billion from 6 July to 22 July 2020. Regarding the input and output table, in order to compute indirect economic losses in the province, this paper uses the input–output table of Jiangxi province in 2012, which was published by Jiangxi Provincial Bureau of Statistics and is the most recent provincial input–output table available for the study period. In addition, for the purpose of analyzing the multi-regional and multi-sectoral indirect economic losses, this paper uses the 2015 China MRIO table in the China Emission Accounts and Datasets (CEADs), which was compiled by Zheng et al. [42]. Because the compilation of MRIO tables needs a large amount of labor, material, and time resources, they are updated around every 5 years or more. Considering the latest obtainable MRIO table when the flood disaster occurred in Jiangxi Province was the 2015 version MRIO table, and the change in the input–output structure is supposed to be gradual, we chose the year 2015 version MRIO table for estimating the indirect economic losses of the flooding. In addition, the 2012 China MRIO table compiled by Liu et al. was also used in the structural decomposition analysis section.

### 3.2. Analysis of Industry-Related Losses within Jiangxi Province

#### 3.2.1. Analysis of the Comprehensive Economic Losses

**Figure 2:**
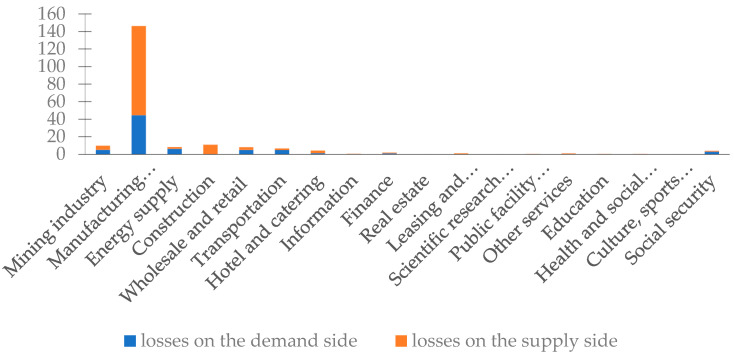
Indirect economic losses on the demand/supply side in Jiangxi province (unit: CNY 100 million).

The detailed results of the indirect economic losses of Jiangxi based on the regional IO table of Jiangxi province in 2012 can be found in Figure 2 and Table 2. As the sum of losses on the demand side and the supply side, the comprehensive economic loss is the overall reflection of the related losses caused by the disaster in the agricultural sector of Jiangxi province. On the whole, the indirect economic losses caused by the agricultural sector to other sectors in the province are 2.08 times the direct economic losses. Of the total indirect losses, the manufacturing sector suffered the worst indirect economic losses, accounting for 70.11% of the total, while the losses of the construction and mining industries accounted for 5.37% and 4.81%, respectively. It is clear that in the comprehensive assessment of economic losses of floods, indirect economic losses should not be ignored, and the losses suffered by different industrial sectors in the province are heterogeneous. It is necessary to carry out an in-depth analysis from the perspective of both the demand and the supply sides.

#### 3.2.2. Analysis of Industry-Related Losses on the Demand Side

The sector suffering the largest indirect economic losses on the demand side was the manufacturing industry, accounting for 59.02% of the total indirect economic losses on the demand side. In addition, the demand side spillover effect I of the manufacturing industry was the highest of the 18 sectors, indicating its closely connection and vulnerability to the influence of other sectors. In addition, the indirect economic losses in industries such as mining, energy supply, wholesale and retail, and transportation were also relatively heavy. In view of the industrial structure, the indirect economic losses of the secondary industry were greater than those of the tertiary industry. Furthermore, the demand side spillover effect II of the agricultural sector was less than 1, at only 0.8747, indicating that the agricultural sector’s ability to influence other sectors is weak, as was also seen for the demand side.

#### 3.2.3. Analysis of Industry-Related Losses on the Supply Side

Because it had the highest spillover effect, the manufacturing industry was the sector with the largest indirect economic losses on the supply side, accounting for 76.45% of the total, followed by the construction industry, which accounted for 8.31%. In terms of industrial structure, the losses of the secondary industry far exceeded those of the tertiary industry, indicating that the secondary industry is more sensitive to floods on the supply side than the tertiary industry. Furthermore, the supply side spillover effect II (1.5316) of the agricultural sector was much higher than the demand side spillover effect II (0.8747), showing that the agricultural sector has a greater influence on the supply side than on the demand side. We can conclude that when the agricultural sector suffers a disaster, regardless of the impacts on the industry itself, the indirect economic losses on the supply side caused by industrial linkages are significantly greater than those on the demand side, reflecting the strong capacity of external spillover, as was seen for the agricultural sector on the supply side.

### 3.3. A Static Analysis of Industry-Related Losses of the Agricultural Sector in Jiangxi Province for China’s Multiple Regions and Sectors

#### 3.3.1. Analysis of Inter-Regional Comprehensive Economic Losses

Based on the 2015 China MRIO table, we calculated indirect economic losses among regions, as shown in Table 3. This shows that the losses of Jiangxi province’s agricultural sector caused inter-regional comprehensive economic losses amounting to CNY 25.5543 billion in 19 sectors in China’s 31 provinces and municipalities. The demand side and supply side related losses accounted for 29.71 percent and 70.29 percent respectively, revealing that the direct economic losses incurred in Jiangxi province are more likely to cause a supply shock for those regions and sectors using agricultural products as inputs. The main explanation for this is that the supply side spillover effect II (2.0122) of the agricultural sector in Jiangxi province is higher than that of the demand side spillover effect II (0.8504). In addition, regarding the national average, the agricultural sector of Jiangxi province has a low demand side spillover effect II and a high supply side spillover effect II, meaning that it easily has a larger influence on the supply side of other sectors in other regions.

#### 3.3.2. Analysis of Inter-Regional Related Losses among Sectors

**Figure 3:**
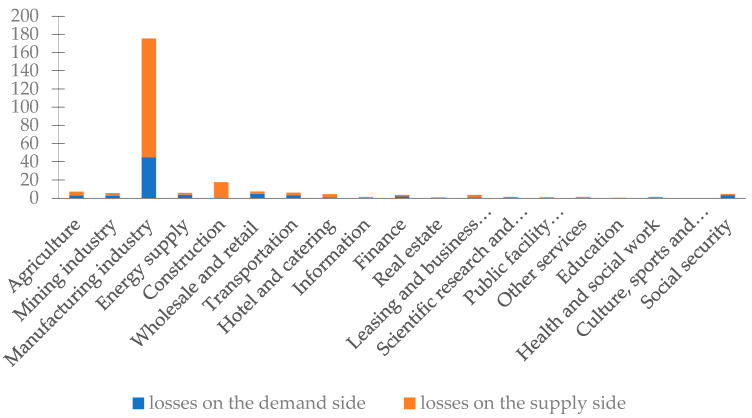
Indirect economic losses on the demand/supply side of various sectors in China (unit: CNY 100 million).

The industry-related losses were divided by departments, as shown in Figure 3 and Table 3. Noticeably, the manufacturing and construction industries (both belong to the secondary industry) were the two sectors with the largest inter-regional comprehensive economic losses; in addition, they are the only two sectors with inter-regional comprehensive economic losses higher than average. In contrast, the education industry and culture, sports, and entertainment industry (both belong to the tertiary industry) were the two sectors with the smallest inter-regional comprehensive economic losses. On the whole, the industry-related losses of various sectors were unbalanced, i.e., the inter-regional comprehensive economic losses of the secondary industry were much higher than those of the tertiary industry. After investigating its underlying causes, it was found that the average spillover effect of the secondary industry was much higher than that of the tertiary industry, and higher than the average, reflecting the importance of the development of other industries to the secondary industry. It also shows that the manufacturing industry is more sensitive to natural disasters such as floods than the related sectors of the service industry.

From the point of view of the demand and supply sides, the losses of the manufacturing industry on both sides were the most serious. In addition, for the demand side, manufacturing was followed by the wholesale and retail trade industry, energy supply industry, and transportation industry; by comparison, in terms of the supply side, this was followed by the construction industry. Thus, due to the high demand side spillover effect I (309.4749) and the high supply side spillover effect I (405.8936) for the manufacturing industry, and the high supply side spillover effect I (108.0466) for the construction industry, the secondary industry has a stronger link and is therefore more vulnerable to floods than the tertiary industry.

#### 3.3.3. Analysis of Related Losses among Regions

**Figure 4:**
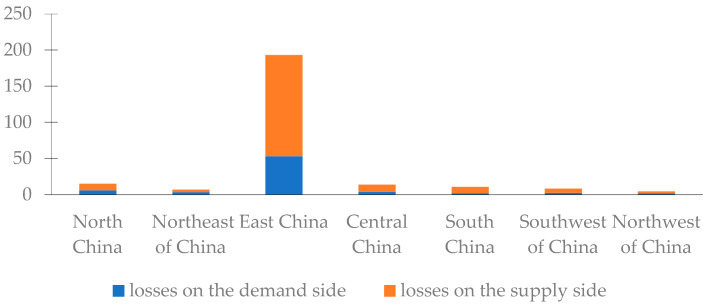
Indirect economic losses on the demand/supply side of various regions in China (unit: CNY 100 million).

The industry-related losses were divided by regions, as shown in Figure 4 and Table 4. Obviously, Jiangxi province’s inter-regional comprehensive economic losses are the largest, amounting to CNY 14.4939 billion. Due to the closer links between industries within Jiangxi province, other sectors in the province are more vulnerable to the impact than those sectors in other provinces. In addition, the related losses in the remaining 30 provinces in China were as high as CNY 11.0604 billion, of which Shandong, Jiangsu, Zhejiang, Henan, Guangdong, Anhui, and other provinces suffered relatively serious damages. Along with Jiangxi province, most of these are located in East China. The three provinces with low inter-regional comprehensive economic losses are located in southwestern and northwestern China. It can be seen that natural disasters such as floods will not only cause indirect economic losses in the local area, but also have a differentiated impact on the related industries in other regions. Furthermore, the severity of the impact is closely related to the geographical location and the industrial relevance to the agricultural sector in Jiangxi province.

Observing the demand and the supply sides, we found that Anhui, Henan, and Jiangsu provinces suffered larger losses for the demand side. In terms of the supply side, Shandong, Jiangsu, and Zhejiang provinces were the three most affected areas suffering serious related losses. Furthermore, Tibet, Qinghai, and Ningxia provinces maintain lower values for demand side and supply side effect I, resulting in weaker links that were far below the average, so the related losses in these three provinces were the lowest on both the demand and supply sides. Moreover, comparing the losses on the demand side with those of the supply side, apart from Jilin, Heilongjiang, Guangxi, and Xinjiang provinces, the direct agricultural economic loss caused by the floods in Jiangxi province is more likely to result in greater losses on the supply side of various regions than on the demand side.

### 3.4. A Dynamic Analysis of Multi-Regional and Multi-Sectoral Related Losses

Based on the 2015 China MRIO table, a static analysis of related losses was carried out above. In this section we calculate the related losses among regions based on the 2012 China MRIO table and compare them with the above calculations, thereby carrying out a dynamic analysis of the differences in the loss data. Using the structural decomposition method, the intrinsic causes of the loss differences were determined, with 2012 as the base period and 2015 as the calculation period. Overall, the total regional economic losses calculated on the basis of 2012 and 2015 MRIO data amounted to CNY 23.0954 billion and CNY 25.5543 billion respectively, with a difference of CNY 2.4586 billion. The differences in the losses on the demand and supply sides were CNY −0.0728 billion and CNY 2.5314 billion, respectively, confirmed by the differences in the spillover effect II (−0.0025, 0.2950) on the demand and supply sides for the agricultural sector in Jiangxi province. This shows that the influence of the agricultural sector in Jiangxi province on the supply side expanded further between 2012 and 2015, while that on the demand side changed only slightly.

#### 3.4.1. Analysis of the Difference in Losses among Sectors

Changes in the inter-sectoral regional comprehensive economic losses show that the related losses of manufacturing and construction (both belong to the secondary industry) expanded significantly, and that the difference in losses between the two sectors is mainly caused by the expansion in supply side losses. During the period of 2012–2015, the supply side spillover effect I of the manufacturing and construction industries increased significantly, and the links strengthened year by year. In addition, the transportation industry, and accommodation and catering industry (both belong to the tertiary industry), suffered the smallest changes in regional comprehensive economic losses, indicating that the industrial structure of the two sectors did not change significantly over 2012–2015, and the spillover effect I changed only slightly. On the whole, the expansion in the losses in the secondary industry between 2012–2015 was significantly larger than that in the tertiary industry, and except for the mining, energy supply, transportation, and accommodation and catering industries, the regional comprehensive economic losses in the sectors showed a further expansion due to the strengthening of industrial linkages year by year.

#### 3.4.2. Analysis of Difference in Losses among Regions

On one hand, the inter-regional comprehensive economic loss differences show that the related losses of Anhui, Henan, Guangdong, and Jiangsu provinces increased. Evidently, Anhui province, which is adjacent to Jiangxi province, underwent the most significant expansion of its related losses, mainly due to the expansion of supply side losses, which is closely associated with the large increase in the supply side spillover effect I in Anhui. On the other hand, the smallest change in related economic losses was in Shanghai, which corresponds to the low difference in the demand side spillover effect I and the low difference in the supply side spillover effect I. Overall, the related economic losses in East China expanded most obviously, and were mainly affected by the supply side, reflecting the increasing trend of links in East China, followed by central and southern China. By comparison, northeast China had the smallest change in related economic losses and even experienced a slight decline, which also reflects the continuous improvement in industrial integration and development capacity in the Yangtze River Economic Belt in recent years.

#### 3.4.3. Structural Decomposition Analysis of Loss Differences

**Figure 5:**
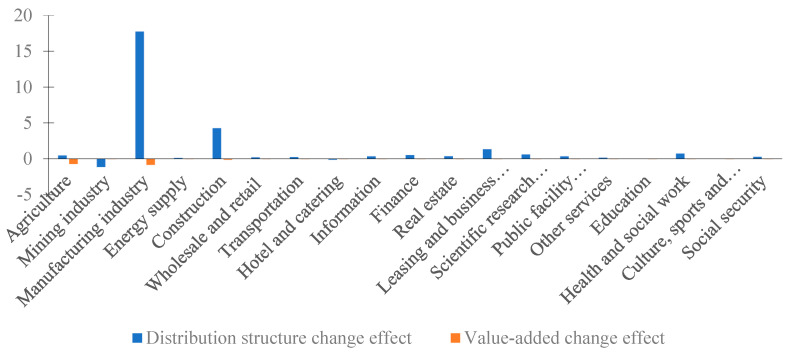
Distribution structure change effect and value-added change effect of various sectors in China (unit: CNY 100 million).

**Figure 6:**
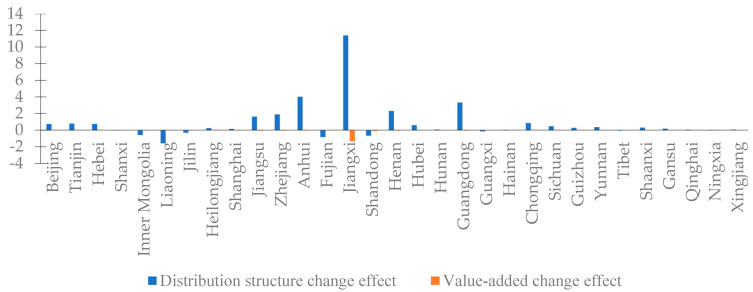
Distribution structure change effect and value-added change effect of various regions in China (unit: CNY 100 million).

Because the difference in regional comprehensive economic losses was mainly caused by the supply side, this section mainly analyzes the difference in supply side losses. We break down the supply side loss changes into the distribution structure change effect and value-added change effect, and divide this into the sectors and regions, as shown in Figure 5 and Figure 6, respectively.

The supply side loss differences show that the distribution structure change effect amounted to CNY 2.7096 billion, and the value-added change effect was only CNY −0.1782 billion. From the perspective of various sectors, the most significant changes in supply side losses were in the manufacturing and construction industries, wherein the distribution structure change effect was CNY 1.7768 billion and CNY 0.4306 billion, respectively. It can be seen that the supply side losses of the manufacturing and construction industries were oriented by their distribution structures. In addition, the value-added change effect of various sectors was negative and the amount was small. From the regional point of view, the supply side losses in East China expanded most obviously, of which the distribution structure change effect was also the largest. In addition to Jiangxi province, the supply side loss changes were also larger in Anhui, Guangdong, and Henan provinces, and were all oriented by the distribution structure change.

We can conclude that there was no significant change in the structure of value added in various sectors between 2012 and 2015, and the changes in the distribution structure that reflect changes in intermediate inputs were the key causes of the difference in supply side losses.

## 4. Conclusions

This article takes the flood disaster in Jiangxi province as the research object, and applies the regional IO model, MRIO model, and dynamic SDA method to investigate the impact of the flooding on the overall and inter-regional economic losses. The results show that the indirect economic losses caused by the flood disaster in the agricultural sector of Jiangxi province are more than twice the direct economic losses in terms of both the intra-regional and inter-regional aspects. Therefore, evaluating economic impacts of floods should be more comprehensive and it is necessary to pay more attention to indirect economic losses. Intra-regional and inter-regional indirect economic losses on the supply side are significantly higher than those on the demand side, indicating that those regions and sectors using agricultural products as inputs are more vulnerable to the impact of losses, and that the agricultural sector on the supply side has a strong capacity of external spillover. In addition to the spillover effect in the agricultural sector, the differentiation of sensing capacity in other sectors also led to differences in indirect economic losses.

Noticeably, spillover effect I of sectors that belong to the secondary industry, including the manufacturing and construction industries, is significantly higher than that of the sectors belonging to the tertiary industry. In addition, location factors also play a significant role in affecting indirect economic losses; sectors within Jiangxi province are more influenced than those outside the province, and the provinces located in eastern China have a higher industrial integration than those located in the southwestern, northwestern, and other distant areas. Thus, in assessing the economic impacts of floods, we should strengthen the analysis from a multi-regional, multi-sectoral perspective. In addition, the dynamic analysis of differences in related losses based on the 2012 and 2015 MRIO table shows that the total regional economic loss difference is positive, and is mainly affected by the expansion of supply side losses. Further structural decomposition of supply side loss differences confirms that the widening of loss differences is mainly caused by changes in distribution structure.

## Figures and Tables

**Table 2:** Indirect economic losses of other sectors in Jiangxi province (unit: CNY 100 million).

Serial Number	Sector	Losses on the Demand Side	Losses on the Supply Side	Comprehensive Economic Losses
1	Mining industry	5.3470	4.7060	10.0530
2	Manufacturing industry	44.8599	101.7458	146.6058
3	Energy supply	6.4493	2.0177	8.4670
4	Construction	0.1755	11.0596	11.2351
	Subtotal of secondary industry	56.8317	119.5291	176.3608
5	Wholesale and retail	5.2265	3.0674	8.2939
6	Transportation	5.0790	1.8074	6.8864
7	Hotel and catering	1.4991	3.0119	4.5110
8	Information	0.3851	0.4413	0.8265
9	Finance	1.3005	0.8491	2.1496
10	Real estate	0.1547	0.2481	0.4028
11	Leasing and business service	0.5105	0.8888	1.3992
12	Scientific research and technical services	0.2690	0.1949	0.4639
13	Public facility management	0.3761	0.2179	0.5940
14	Other services	0.6217	0.6577	1.2794
15	Education	0.3288	0.3707	0.6995
16	Health and social work	0.0661	0.6332	0.6993
17	Culture, sports and entertainment	0.1279	0.2469	0.3748
18	Social security	3.2296	0.9238	4.1535
	Subtotal of tertiary industry	19.1748	13.5590	32.7338
	Total indirect economic losses	76.0063	133.0882	209.0945

**Table 3:** Related losses of various sectors in China (unit: CNY 100 million).

Serial Number	Sector	Losses on the Demand Side	Losses on the Supply Side	Comprehensive Economic Losses
1	Agriculture	3.3645	4.1999	7.5644
	Subtotal of primary industry	3.3645	4.1999	7.5644
2	Mining industry	2.7417	3.1830	5.9246
3	Manufacturing industry	44.9852	130.7525	175.7376
4	Energy supply	4.0481	2.0440	6.0921
5	Construction	0.1612	17.7282	17.8893
	Subtotal of secondary industry	51.9361	153.7076	205.6437
6	Wholesale and retail	5.1460	2.5585	7.7045
7	Transportation	3.7063	2.7480	6.4543
8	Hotel and catering	1.1305	3.6250	4.7556
9	Information	0.6928	0.9882	1.6810
10	Finance	2.1842	1.6879	3.8721
11	Real estate	0.3738	0.7786	1.1524
12	Leasing and business service	1.1864	2.7144	3.9008
13	Scientific research and technical services	0.5361	1.2121	1.7482
14	Public facility management	0.5388	0.8110	1.3498
15	Other services	0.7467	1.0230	1.7697
16	Education	0.3905	0.4241	0.8145
17	Health and social work	0.1369	1.6082	1.7450
18	Culture, sports and entertainment	0.1810	0.3301	0.5110
19	Social security	3.6677	1.2081	4.8758
	Subtotal of tertiary industry	20.6177	21.7171	42.3348
	Total indirect economic losses	75.9183	179.6246	255.5429

**Table 4:** Related losses of various regions in China (unit: CNY 100 million).

Serial Number	Region	Losses on the Demand Side	Losses on the Supply Side	Comprehensive Economic Losses
1	Beijing	1.4155	2.3265	3.7421
2	Tianjin	1.1340	2.1373	3.2714
3	Hebei	1.6707	2.1470	3.8177
4	Shanxi	0.7552	0.8083	1.5635
5	Inner Mongolia	1.3112	1.7947	3.1060
	Subtotal of North China	6.2867	9.2139	15.5006
6	Liaoning	1.0617	1.5573	2.6190
7	Jilin	1.4411	0.5040	1.9451
8	Heilongjiang	1.4420	1.3707	2.8127
	Subtotal of Northeast China	3.9448	3.4320	7.3768
9	Shanghai	1.3699	1.9448	3.3147
10	Jiangsu	2.8470	8.2146	11.0617
11	Zhejiang	1.2268	7.9268	9.1536
12	Anhui	2.3029	6.5437	8.8467
13	Fujian	0.9418	1.1379	2.0797
14	Jiangxi	42.9173	102.0216	144.9389
15	Shandong	1.9628	12.1305	14.0934
	Subtotal of East China	53.5685	139.9200	193.4885
16	Henan	2.5466	6.4558	9.0024
17	Hubei	0.3788	1.8696	2.2484
18	Hunan	1.4095	1.5860	2.9954
	Subtotal of Central China	4.3349	9.9113	14.2462
19	Guangdong	1.3789	7.5787	8.9576
20	Guangxi	0.8686	0.7159	1.5845
21	Hainan	0.2939	0.4103	0.7042
	Subtotal of South China	2.5413	8.7050	11.2463
22	Chongqing	0.6271	2.3517	2.9789
23	Sichuan	0.8848	1.6296	2.5144
24	Guizhou	0.7583	0.6677	1.4259
25	Yunnan	0.6596	1.1356	1.7952
26	Tibet	0.0154	0.0175	0.0330
	Subtotal of Southwest China	2.9452	5.8022	8.7473
27	Shaanxi	1.1337	1.4095	2.5432
28	Gansu	0.3617	0.5487	0.9104
29	Qinghai	0.0572	0.1313	0.1885
30	Ningxia	0.1586	0.1635	0.3221
31	Xingjiang	0.5857	0.3873	0.9730
	Subtotal of Northwest China	2.2968	2.6402	4.9371
	Total loss	75.9183	179.6246	255.5429

## Data Availability

The data presented in this study are available on request from the corresponding author.
